# Role of SGK1 for fatty acid uptake, cell survival and radioresistance of NCI-H460 lung cancer cells exposed to acute or chronic cycling severe hypoxia

**DOI:** 10.1186/s13014-016-0647-1

**Published:** 2016-06-01

**Authors:** Johann Matschke, Elisa Wiebeck, Sebastian Hurst, Justine Rudner, Verena Jendrossek

**Affiliations:** Institute of Cell Biology (Cancer Research), University of Duisburg-Essen, University Hospital Essen, Virchowstrasse 173, 45122 Essen, Germany

**Keywords:** Hypoxia, Fatty acid uptake, Ionizing radiation, SGK1, Unsaturated fatty acids, Oleic acid, Radioresistance, SCD1

## Abstract

**Background:**

Unsaturated fatty acids (FA) are required for cancer cell growth. In normoxia cells can generate unsaturated FA from saturated stearic and palmitic acid by desaturation. However, since the desaturation step is oxygen-dependent hypoxic cancer cells display an increased dependence on the uptake of unsaturated FA. Up to now the mechanism of increased FA uptake in hypoxia is largely unknown. Here we aimed to study the role of human serum and glucocorticoid-inducible kinase (SGK1) in the regulation of FA uptake in cancer cells exposed to acute or chronic cycling hypoxia and explore its use as target for the radiosensitization of hypoxic cancer cells.

**Methods:**

The effect of SGK1-inhibition (GSK650394) on NCI-H460 lung adenocarcinoma cells exposed to normoxia, acute or chronic cycling hypoxia was analyzed under standard and serum-deprived conditions by short-term proliferation, apoptosis and cell death assays. The impact of SGK1-inhibition on radiation sensitivity was determined by standard colony formation assays. The effect of GSK650394 on FA uptake was quantified by measuring intracellular accumulation of fluorescent FA (C1-BODIPY®-C12).

**Results:**

Exposure to acute or chronic cycling hypoxia was associated with up-regulated expression of SGK1 in NCI-H460 cells, increased uptake of FA from the culture medium, and increased sensitivity to serum deprivation. Survival of serum-deprived hypoxic NCI-H460 cells was rescued by the addition of the unsaturated FA, oleic acid, whereas the saturated FA, palmitic acid was highly toxic to the hypoxic cancer cells. Interestingly, SGK1 inhibition abrogated the rescue effect of oleic acid in serum-deprived hypoxic cancer cells and this effect was associated with a reduction in FA uptake particularly in anoxia-tolerant cancer cells exposed to severe hypoxia. Finally, SKG1 inhibition decreased long-term survival and potently sensitized the parental and anoxia-tolerant NCI-H460 cells to the cytotoxic effects of ionizing radiation in normoxia as well as the anoxia-tolerant cancer cells in severe hypoxia.

**Conclusions:**

Our data suggest that SGK1 plays a role in the regulation of FA uptake that becomes essential under conditions of acute or chronic cycling hypoxia. We assume that SGK1 may represent a promising therapeutic target for the eradication of hypoxic cancer cells.

## Background

Tumor hypoxia is a common feature of solid human tumors and constitutes a major obstacle to successful radiotherapy [1, 2]. Tumor hypoxia, which is characterized by reduced tissue oxygenation, arises either as acute hypoxia resulting for example from a disturbed microcirculation or reversible occlusion of tumor blood vessels, or as chronic hypoxia for example as a result of the increased oxygen diffusion distance to the capillary in rapidly growing tumors [3–9]. Acute and chronic hypoxia as well as moderate and severe hypoxia can coexist in the same tumor; thereby tumors are exposed to a continuum of varying high and low oxygen partial pressures [3, 6, 8, 10, 11]. Tumor hypoxia can also be highly dynamic with cycles of hypoxia and intermittent re-oxygenation termed cycling hypoxia [4, 10, 12, 13]. Tumor hypoxia promotes phenotypic heterogeneity, malignant progression and clonal evolution of chemotherapy and radiotherapy resistance, among others by hypoxia-induced genomic instability and adaptive changes in cell metabolism [8, 14–17].

One major metabolic pathway affected by hypoxia is the lipid metabolism [18, 19]. Cell growth, proliferation and survival involve the formation of new membranes, which require the production of new lipids with an appropriate molecular composition. The process by which acetyl coenzyme A is converted to fatty acids (FA) is named lipogenesis [18, 20]. The basic step in this pathway is the oxidation of saturated FA into mono-unsaturated FA that serve as a major FA species in mammalian membranes, cell signaling and as a pool of metabolic energy, respectively [18, 21]. The oxidation step is catalyzed by stearoyl-CoA desaturase (SCD1), a FA desaturase expressed in most adult tissues. The enzyme is responsible for maintaining the balance of saturated FA and unsaturated FA by converting absorbed exogenous and endogenously synthesized saturated FA into (mono)-unsaturated FA [18, 22]. It was generally assumed that tumor cells synthesize most of the non-essential FA autonomously. However, a recent study revealed that the provision of unsaturated FA for cancer cell growth is highly dependent on oxygen supply to ensure the availability of sufficient unsaturated FA [23]. In normoxia, high FA synthesis emerges from glucose metabolites [18, 24]. During this process, saturated FA are formed, desaturated by SCD1, and subsequently elongated. However, hypoxia leads to inhibition of the oxygen-consuming enzyme SCD1 [18, 22]. The resulting impairment of the desaturation process leads to increased dependence of hypoxic cancer cells on the uptake of unsaturated FA from the environment for maintaining cellular processes such as membrane formation and signaling [19, 23]. A similar dependency on the uptake of unsaturated FA is achieved in normoxic cells by inhibition of SCD1 [25, 26].

Up to now, the molecular mechanisms that mediate and control the uptake of FA from the environment are widely unknown. But a recent study performed in yeast suggested that the expression of YPK1, the yeast ortholog of human serum and glucocorticoid-inducible kinase SGK1, is linked to FA uptake and cell growth in hypoxia [27]. SGK1 is a serine/threonine-kinase with a highly conserved kinase structure that is ubiquitously expressed in human tissues and activated by growth factors and hormones via the PI3-Kinase pathway [28–34]. Since our preliminary data suggested a prominent up-regulation of SGK1 in hypoxia we speculated that in addition to its reported role in the activation of various ion channels and nutrient transporters SGK1 might regulate the FA uptake in cancer cells.

The present study was designed to investigate a dependency of NCI-H460 lung adenocarcinoma cells exposed to acute severe hypoxia (0.2 % O_2_) on the uptake of (unsaturated) FA present in serum and to evaluate the role of SGK1 in regulating this process. In addition, we were interested whether chronic exposure of NCI-H460 lung cancer cells to 25 cycles of anoxia (48 h 0.1 % O_2_)/re-oxygenation stress (120 h 20 % O_2_) changes the metabolic dependencies of these “anoxia-tolerant cells” [16]. Finally, we aimed to explore the potential of SGK1-inhibition to increase the cytotoxic activity of ionizing radiation in parental and anoxia-tolerant cancer NCI-H460 cells.

## Materials and methods

### Reagents and antibodies

GSK650394 was purchased from Tocris (Bristol, UK). Fluorescent FA (C_1_-BODIPY® 500/510 C_12_) were purchased from Thermo Scientific (MA, US). Propidium iodide (PI) was from Gibco/Life Technologies (Carlsbad, CA). Stock solutions of GSK650394 were made with DMSO. All other chemicals were purchased from Sigma-Aldrich (St. Louis, MO) if not otherwise stated.

The following antibodies were used for western blot analysis: anti-goat SGK1 (C-20) and anti-rabbit Calnexin (H-70) from Santa Cruz Biotechnology (Heidelberg, Germany).

### Cell lines and treatment

The lung adenocarcinoma cell line NCI-H460 was obtained from ATCC (Bethesda, MD). NCI-H460 lung adenocarcinoma cells with tolerance to cycling severe hypoxia/re-oxygenation stress (“anoxia-tolerant cells”) were generated by exposure to 25 cycles of severe hypoxia (48 h, 0.1 % O_2_) and re-oxygenation (120 h air plus 5 % CO_2_ referred as 20 % O_2_) as described recently [16]. Cells were routinely grown in RPMI 1640 medium supplemented with 10 % (v/v) fetal calf serum (Gibco/Life Technologies, Carlsbad, CA) and maintained in a humidified incubator at 37 °C and 5 % CO_2_ (referred to as “normoxia“or “normoxic conditions“, Nx). Under hypoxic conditions cells were grown in a humidified hypoxia workstation (In vivo 400, Ruskinn Technology Ltd., Bridgend, Great Britain) at 37 °C, 0.2 % O_2_, and 5 % CO_2_ (referred to as “hypoxia” or “hypoxic conditions“, Hx).

Serum withdrawal experiments started 4 h after seeding by medium exchange to low serum media (0.5 % FCS). Cells were treated with irradiation (0-10 Gy) without or with SGK1 inhibitor (0–100 μM GSK650394) 24 h (h) after seeding for up to 72 h. Lipid supplementation (50 μM oleic acid (OA) or 50 μM palmitic acid (PA)) was performed 24 h after seeding for up to 72 h. For combined treatment, drugs were added 2 h before irradiation. For treatment under hypoxic conditions, cells were transferred to the hypoxic chamber 2 h before drug treatment. 0.1 % DMSO was used as a solvent control in all treatment experiments. Irradiation was performed at room temperature with an X-ray machine (Precision X-Ray Inc., North Branford, CT) operated at 320 kV, 12.5 mA with a 1.65 mm Al filter, at a distance of 50 cm and a dose rate of 3.71 Gy/min. Cells were returned to the incubator immediately after exposure to ionizing radiation.

### qRT-PCR analysis

cDNA was synthesized from 1 μg of total RNA using QuantiTect® Reverse Transcription Kit (Qiagen, Hilden, Germany) according to the manufacturer’s protocol. Specific primers were synthesized based on available sequences for each listened gene. Primer sets for quantitative real-time PCR (qRT–PCR) were designed with the program Primer 3 [35]. To exclude cross-reaction of primers with the genes the sequence of interest was compared with a database (Blast 2.2, U.S. National Centre for Biotechnology Information, Bethesda, MD) and all primers used in our study were intron-spanning. PCR products were 300–400 bp in size. We used published β2-microglobulin (B2M) primer sequences as housekeeping gene [36]. qRT–PCR and cycling conditions were performed using specific oligonucleotide primers (*B2M* forward: TGCTGTCTCCATGTTTGATGTATCT; reverse, TCTCTGCTCCCCACCTCTAAGT; *SGK1*: forward, TGTTCATGGGAAACTCAATCTG; reverse, TTGCATGCATAGGAGTTATTGG) using qPCR kit for SYBR® Green I, 6-Carboxyl-X-Rhodamine (ROX) (Eurogentec, Cologne, Germany) according to the manufacturers protocol. Reactions were carried out on an ABI Prism 7900HT using MicroAmp Optical 96 well Reaction plate (Applied Biosystems by Life Technologies, Bleijswijk, Netherlands) and BIO-RAD PCR Sealers Microseal “B” Film Adhesive seal (optically clear; BIORAD, Munich, Germany). Melting curves were obtained after each PCR run and showed single PCR products. cDNAs were run in triplicate, without reverse transcriptase and no-template controls were run in duplicates. Expression levels for the genes of interest and for the housekeeping gene *B2M* were measured in three independent PCR runs. Expression ratios were calculated using the geometric mean expression of the housekeeping gene *B2M* to normalize the expression data for the genes of interest according to the 2^-ΔΔCt^ – method as described by others [37].

### Western blot analyis

Cells were lysed in 200 μl of lysis buffer containing 50 mM Hepes (pH 7.5), 150 mM NaCl, 1 % Triton X-100, 1 mM EDTA, 10 mM sodium pyrophosphate, 10 mM NaF, 2 mM Na3VO4, 100 mM PMSF, 5 μg/ml aprotinin, 5 μg/ml leupeptin, and 3 μg/ml pepstatin. Proteins were separated by sodium dodecyl sulfate-polyacrylamide gel electrophoresis (SDS-PAGE) under reducing conditions and transferred onto polyvinylidene fluoride (PVDF) membranes (Roth, Karlsruhe, Germany). Blots were blocked in TBS buffer containing 0.05 % Tween 20 and 5 % non-fat dried milk for 1 h at room temperature. The membrane was incubated overnight at 4 °C with the respective primary antibodies. The secondary antibody was incubated for 1 h at room temperature. Detection of antibody binding was performed by enhanced chemiluminescence (ECL Western Blotting Analysis System; GE Healthcare/Amersham Biosciences, Freiburg, Germany). Equal protein loading was verified by calnexin-staining. Densitometric analysis was performed using ImageJ 2.00, National Institutes of Health, Bethesda, MD).

### Determination of fatty acid uptake

The uptake of FA was quantified by using fluorescent FA analog C_1_-BODIPY® 500/510 C_12_. In brief, fluorescent FA (5 μM) were added 24 h after treatment to serum-free media. We quenched the fluorescence of FA in media by adding trypan blue (0.33 mM) to the media. The uptake of fluorescent FA was measured for 1 h, in 1 min intervals, at 37 °C spectrophotometrically at 485/528 nm.

### Flow cytometry analysis

For quantification of apoptotic DNA fragmentation (subG1 population), cells were incubated for 30 min at room temperature with a staining solution containing 50 μg/ml PI in a hypotonic citrate buffer 0.1 % (w/v) sodium citrate and 0.05 % (v/v) Triton X-100 and subsequently analyzed by flow cytometry (Flow Cytometer BD Accuri™ C6, BD Bioscience, Heidelberg, Germany; FL-2) [38].

Cell death was quantified by flow cytometry (Flow Cytometer BD Accuri™ C6, Becton Dickinson) of PI–stained cells. Cells were incubated for 30 min in the dark with PI (0.01 mg/ml) in PBS and measured within 1 h (Flow Cytometer BD Accuri™ C6, BD Bioscience, Heidelberg, Germany; FL-2).

### Cell viability assay

Cells were washed with PBS (1x), fixed with Glutaraldehyde (0.1 % in PBS), and stained with crystal violet (0.1 % in PBS). The dye was released by TritonX-100 (0.2 % in PBS) and measured spectrophotometrically at 540 nm as described elsewhere [39].

### Colony formation assay

Clonogenic cell survival in response to the respective treatments was determined comparing cells cultured under normoxic and severely hypoxic conditions. For treatment in normoxia (Nx), exponentially growing cells were seeded in tissue culture flasks, incubated under standard culturing conditions (20 % O_2_, 5 % CO_2_, 37 °C) and irradiated 24 h later (0 to 5 Gy) without or with drug treatment. Drug treatment was performed 2 h prior to irradiation treatment. For treatment in hypoxia (Hx), tissue culture flasks of exponentially growing cells were transferred into the hypoxic chamber 2 h prior to drug treatment and 4 h prior to irradiation, respectively. After completion of treatment, cells were incubated for 24 h under standard or hypoxic conditions. Cells were then collected (0.05 % Trypsin, 0.01 EDTA), washed, plated to 6 well plates at densities of 200 to 3200 cells per well, and subsequently incubated for 10 days under standard normoxic conditions.

For quantification of colony formation, cells were fixed in 3.7 % formaldehyde and 70 % ethanol, stained with 0.05 % Coomassie blue and colonies of at least 50 cells were counted by GelCount (Oxford Optronix, Oxfordshire, Great Britain). The plating efficiency and surviving fraction (SF) were calculated as described elsewhere [40].

### Statistics

Data represent mean values of at least 3 independent experiments ± standard deviation (SD). Data analysis was performed either by unpaired students *t*-test or by two-way ANOVA test (Prism5TM software, Graph Pad Inc., La Jolla, US states) using parametric methods and employing Bonferroni multiple comparison post-test where appropriate. *P*-values ≤ 0.05 were considered significant.

## Results

### SGK1 expression increases in severe hypoxia and promotes FA uptake

**Fig. 1:**
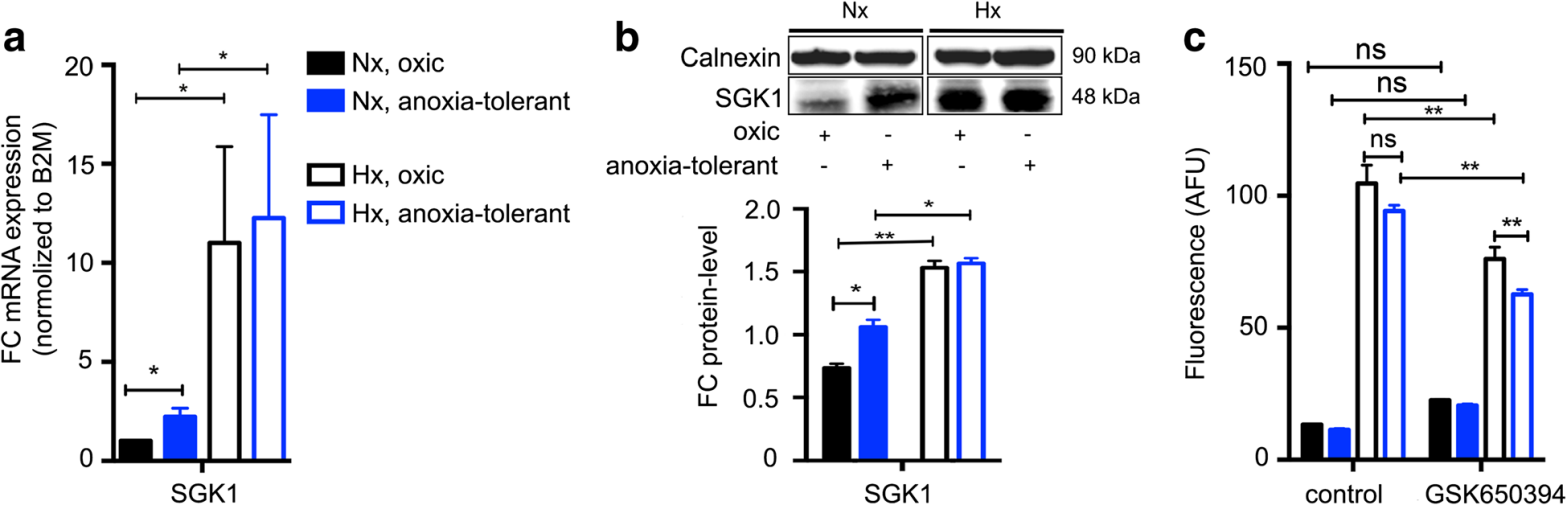
SGK1 expression increases under hypoxia and regulates fatty acid uptake. NCI-H460 cells were exposed to acute (24 h 0.2 % O_2_) or chronic cycling hypoxia (25 cycles of severe hypoxia (48 h, 0.1 % O_2_) and re-oxygenation (20 % O_2_)). **a** The expression of SGK1 in NCI-H460 cells was determined using qRT-PCR analysis. Anoxia-tolerant cells displayed a significant increase in the expression of SGK1 compared to oxic control cells under normoxia. Both cell lines showed a pronounced up-regulation of SGK1 mRNA in severe hypoxia. **b** Western blot analysis of SGK1 protein expression. Upper panel shows representative Western blots of SGK1 and calnexin expression levels. Lower panel shows densitometric evaluation of SGK1 levels normalized to calnexin expression under the respective conditions. SGK1 protein levels were significantly increased in anoxia-tolerant cells compared to oxic control cells in normoxia as well as in response to severe hypoxia in both cell lines. **c** The impact of SGK1 inhibition by GSK650394 (40 μM for 24 h) on the uptake of FA was quantified under normoxic and severely hypoxic conditions in parental and anoxia-tolerant NCI-H460 cells by using the fluorescent FA analogue C_1_-BODIPY® 500/510 C_12_. SGK1-inhibition by GSK650394 did not significantly alter FA-uptake in normoxia, but significantly decreased the uptake of FA in severe hypoxia in both cell lines. Data show representative Western blots or mean values ± SD are shown, *n* = 3. (* *p* ≤ 0.05, ** *p* < 0.01; unpaired students *t*-test)

To examine deregulated expression of SGK1 in NCI-H460 cells exposed to acute or chronic cycling hypoxia we determined the SGK1 mRNA and protein expression levels of parental (“oxic”) NCI-H460 cells and anoxia-tolerant NCI-H460 control cells exposed to normoxia (20 % O_2_) or acute severe hypoxia (0.2 % O_2_) by quantitative RT-PCR (qRT-PCR) or western blot (WB). As shown in Fig. 1a, anoxia-tolerant cells were characterized by significant higher SGK1 mRNA expression levels under normoxic conditions. However, exposure to acute severe hypoxia led to a significant increase in SGK1 expression in both cell lines pointing to a potential role of SGK1 in the response of the lung cancer cells to acute severe hypoxia (Fig. 1a). Our data were supported by Western blot analysis showing increased expression of SGK1 in the anoxia-tolerant cells compared to oxic control cells in normoxia as well as an increased expression of SGK1 in both cell lines on exposure to hypoxia (Fig. 1b).

Next we aimed to verify our hypothesis on a role of SGK1 in FA uptake. For this, we measured the effect of treatment with a specific SGK1 inhibitor (GSK650394) on the uptake of fluorescently labeled FA in oxic and anoxia-tolerant NCI-H460 cells exposed to normoxic and severely hypoxic conditions. We chose a concentration of 40 μM on the basis of LC50 values of 40 to 100 μM obtained in cell-based in vitro assays by others [41]. SGK1 inhibition did not significantly alter the uptake of FA under normoxic conditions in both cell lines, though a slight increase in FA uptake on treatment with GSK650394 was observed (Fig. 1c). However, exposure to severe hypoxia triggered a significant increase in the uptake of FA in both cell lines to a similar extent. Importantly, the hypoxia-induced increase in FA uptake was significantly reduced in both cell variants upon treatment with the SGK1 inhibitor (Fig. 1c). These observations implicate that SGK1 plays a role in the regulation of FA uptake in severe hypoxia.

### Acute and chronic cycling hypoxia leads to dependence on unsaturated FA whereas saturated FA exert toxic effects

**Fig. 2:**
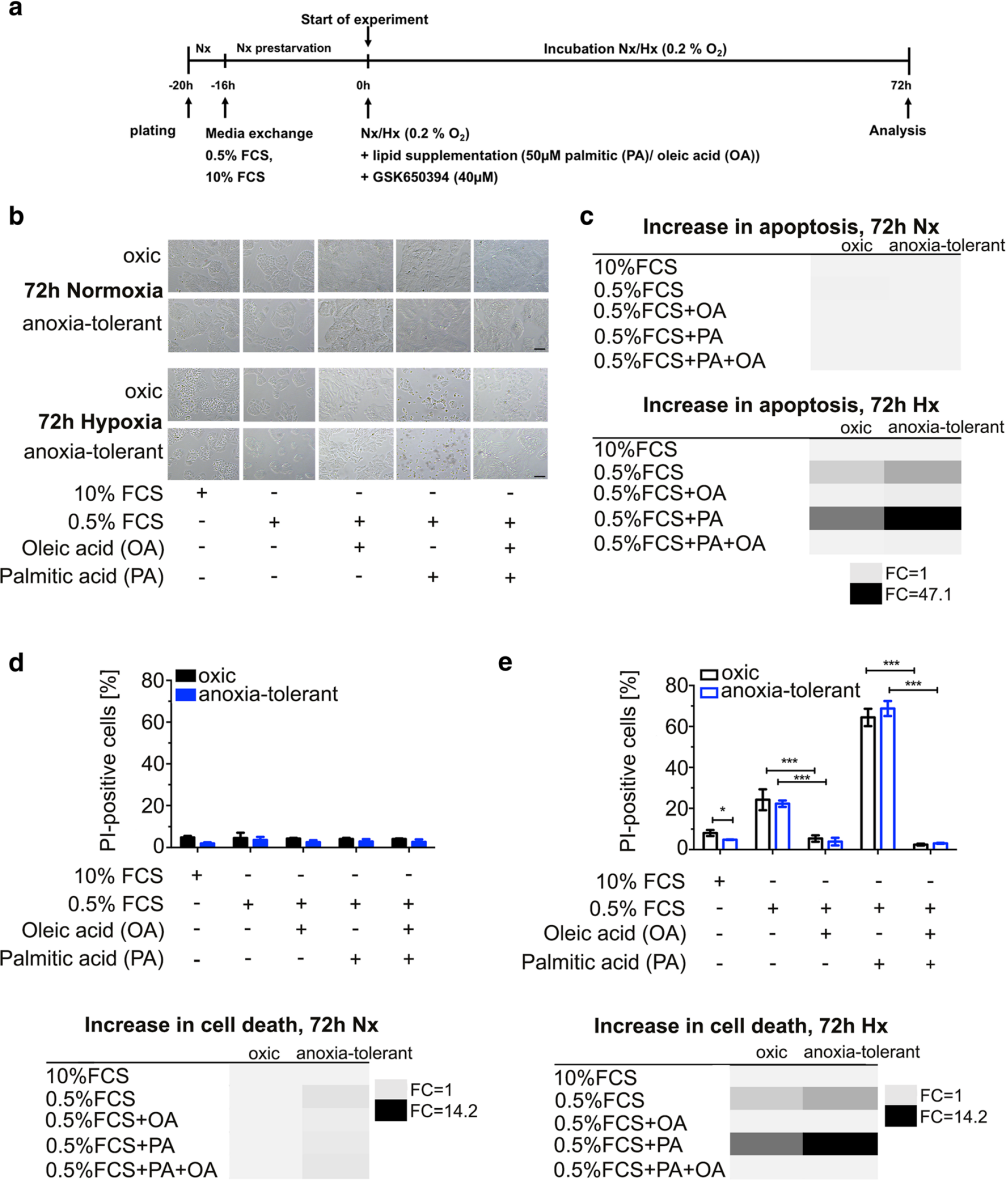
Hypoxia leads to dependence on unsaturated FA whereas saturated FA exert toxic effects. To analyze the influence of serum deprivation and lipid supplementation on the survival of oxic and anoxia-tolerant NCI-H460 cells, cells were cultured for 72 h in standard medium (10 %FCS) or serum-deprived medium (0.5%FCS) without or with additional supplementation of saturated (50 μM palmitic acid, PA) or unsaturated FA (50 μM oleic acid, OA). **a** Schematic representation of the experimental timeline. **b** Representative light microscopic pictures of NCI-H460 cells 72 h after treatment under the indicated conditions in normoxia (20 % O_2_) or severe hypoxia (0.2 % O_2_). Scale bar = 10 μM. **c** Representation of the increase in apoptosis as heat map (fold changes; FC). Apoptosis induction was quantified 72 h after treatment by measuring DNA fragmentation upon staining of the cells with PI in a hypotonic citrate buffer (subG1 fraction). **d**, **e** Cell death was quantified 72 h after treatment by flow cytometry upon staining the cells with PI under normoxic (**d**) or severely hypoxic (**e**) conditions. The increase in cell death levels by a particular treatment is visualized in a heat map representing fold changes (**d** lower panel, **e** lower panel). Almost no effect on apoptosis induction (**c**) or cell death levels (**d**) was observed in both cell lines under indicated treatment conditions in normoxia. Serum deprivation and supplementation of palmitic acid induced apoptosis and increased cell death levels in both cell lines in severe hypoxia, particularly in anoxia-tolerant NCI-H460 cells (**c**, **e**). Supplementation of oleic acid abolished the increase in apoptosis and cell death induced by serum-deprivation without or with PA-supplementation in both cell lines under severe hypoxia (**c**, **e**). Mean values ± SD are shown, *n* = 3. (* *p* ≤ 0.05, *** *p* ≤ 0.001; two- way ANOVA with Bonferroni post-test)

Next we compared the role of FA for short-term cell survival of parental (oxic) and anoxia-tolerant NCI-H460 cancer cells in normoxic and severely hypoxic conditions. For this, survival of oxic and anoxia-tolerant cells was compared in standard medium versus serum-deprived culturing conditions. While serum deprivation in normoxia had no effect on survival of oxic control cells compared to culturing in standard medium (Fig. 2b-d) anoxia-tolerant cells turned out to be slightly sensitive to serum deprivation in normoxia (Fig. 2d, lower panel). In contrast, serum deprivation combined with severe hypoxia led to a pronounced increase in the levels of apoptosis and cell death in both cell variants (Fig. 2b, c, e). When comparing the fold-changes in apoptosis or cell death upon serum withdrawal, the anoxia-tolerant cells were more sensitive to serum deprivation in severe hypoxia compared to the oxic control cells (Fig. 2c, e). Further, we tested whether the dependence of oxic and anoxia-tolerant cells on serum may be mediated by SGK1 and therefore analyzed the mRNA expression levels of SGK1 under serum-deprived conditions. These data showed a significant decrease in hypoxia-induced SGK1-expression as a response to serum deprivation in hypoxia (oxic cells: FC = 3.9 ± 0.8 versus 11.0 ± 4.8 in full medium; anoxia-tolerant cells, Hx: FC = 5.2 ± 0.8 versus 12.3 ± 5.2 in full medium), whereas serum-deprived condition had no significant effect on mRNA SGK1 expression levels in normoxia. To analyze whether the toxic effect of serum deprivation was due to a deficiency in provision of unsaturated FA, we tested whether we could increase cell survival by supplementation of the medium with unsaturated FA (oleic acid), saturated FA (palmitic acid), or the combination of both (Fig. 2a). While supplementation of serum-deprived NCI-H460 cells with oleic acid or palmitic acid alone or in combination did not alter their survival in normoxia (Fig. 2b, c, d), the addition of oleic acid reduced apoptosis induction and rescued NCI-H460 cancer cells from the toxic effects of serum deprivation in severe hypoxia (Fig. 2c, e). In contrast supplementation of serum-deprived hypoxic NCI-H460 cancer cells with palmitic acid, a saturated FA, was even more toxic to hypoxic cancer cells than serum deprivation alone (Fig. 2b, c, e). However, the addition of palmitic acid was not able to counteract the rescue effect of oleic acid under serum-deprived conditions (Fig. 2b, c, e). Altogether these findings corroborate the importance of supplementation with unsaturated FA for survival of cancer cells in (severe) hypoxia and highlight a particular dependence of anoxia-tolerant cells on oleic acid supplementation.

### SGK1 inhibition abrogates the rescue effect of oleic acid-supplementation

**Fig. 3:**
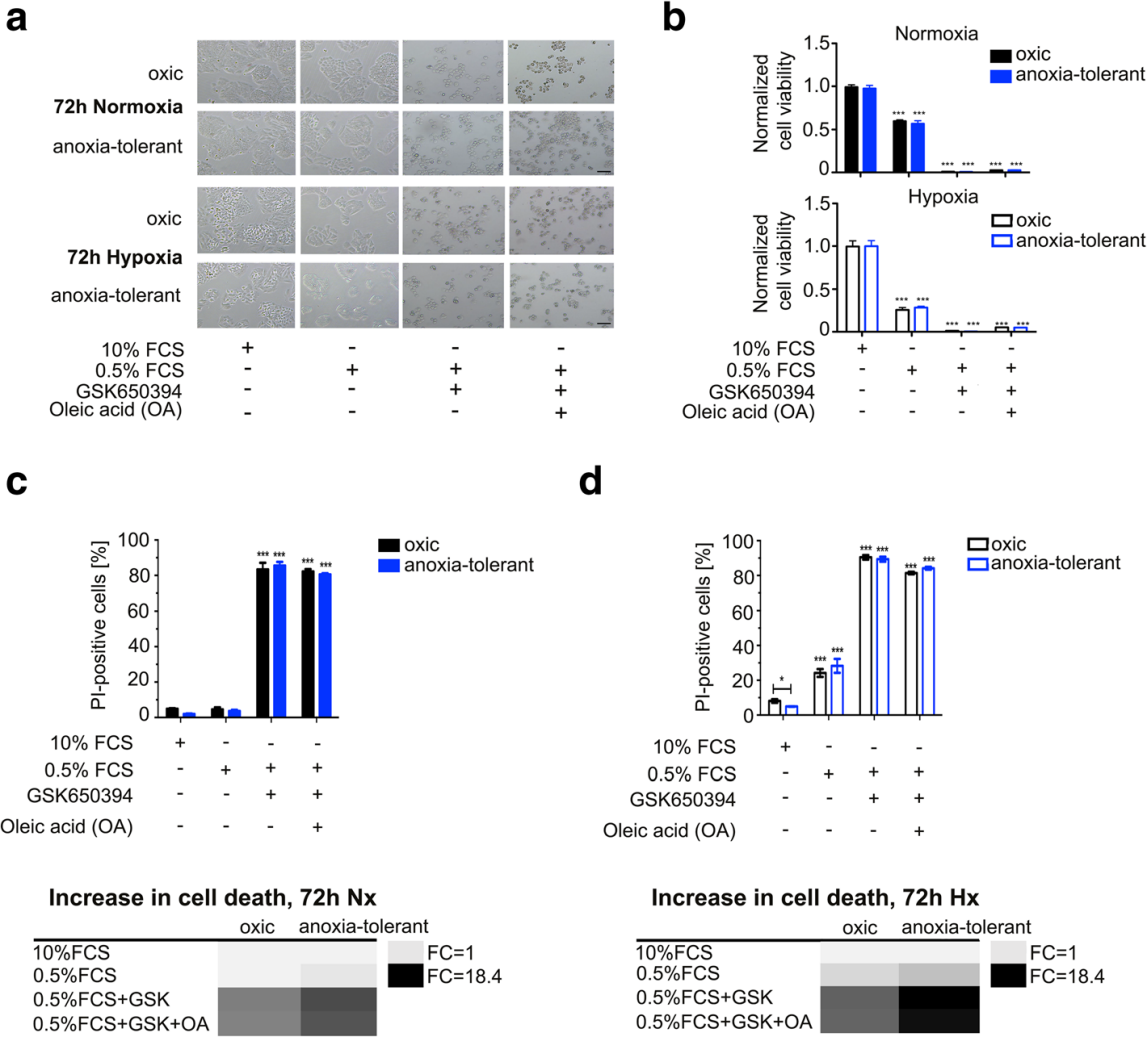
SGK1 inhibition abrogates the rescue effect of oleic acid. Oxic and anoxia-tolerant NCI-H460 cells were treated with the SGK1 inhibitor GSK650394 (40 μM) under serum-deprived conditions with or without supplementation of oleic acid (5 mM) for 24 h under normoxic (20 % O_2_) or severely hypoxic conditions (0.2 % O_2_). **a** Representative light microscopic pictures of oxic and anoxia-tolerant NCI-H460 cells 72 h after the indicated treatment under normoxic or severely hypoxic conditions. Scale bar = 10 μM. **b** Number of viable cells determined by crystal violet assay 72 h after the indicated treatments in normoxia (upper panel) or severe hypoxia (lower panel). **c**, **d** Cell death was quantified 72 h after treatment by flow cytometry as described in Fig. 2. The increase in cell death levels by a particular treatment was visualized in a heat map representing fold changes in normoxia (**c**, lower panel) and severe hypoxia (**d**, lower panel). SGK1 inhibition by GSK650394 decreased cell viability and increased the amount of dead cells in both cell lines under normoxic (b upper panel, **c**) and severely hypoxic conditions (**b** lower panel, **d**). Cell death induction was more prominent in anoxia-tolerant NCI-H460 cells (**c** lower panel, **d** lower panel). SGK1 inhibition abrogates the rescue effect of supplementation with oleic acid (50 μM) on serum deprivation induced cell death under severely hypoxic conditions (**b**-**d**). Mean values ± SD are shown, *n* = 3. (* *p* ≤ 0.05, *** *p* ≤ 0.001; two-way ANOVA with Bonferroni post-test)

To investigate a potential role of SGK1 for cell survival under the diverse conditions, the SGK1 inhibitor GSK650394 was used according to the treatment schedule depicted in Fig. 2a. Inhibition of SGK1 in serum-deprived cells resulted in a significant decrease in proliferation and increase of cell death levels under normoxic (Fig. 3a, b, c) and severely hypoxic conditions (Fig. 3a, b, d). To verify the suggested role of SGK1 in FA uptake we tested whether SGK1-inhibition affects the beneficial effect of oleic acid supplementation in serum-deprived cancer cells. Interestingly, inhibition of SGK1 abrogated the rescue effect of oleic acid supplementation on cell death induced by serum deprivation in severe hypoxia (Fig. 3a, b, d). These results clearly demonstrate a general pro-survival effect of SGK1 in oxic and anoxia-tolerant NCI-H460 cancer cells under normoxic and severely hypoxic conditions and support a contribution of SGK1 in the regulation of FA uptake in severe hypoxia.

### SGK1 inhibition decreases long-term survival and sensitizes to ionizing radiation

**Fig. 4:**
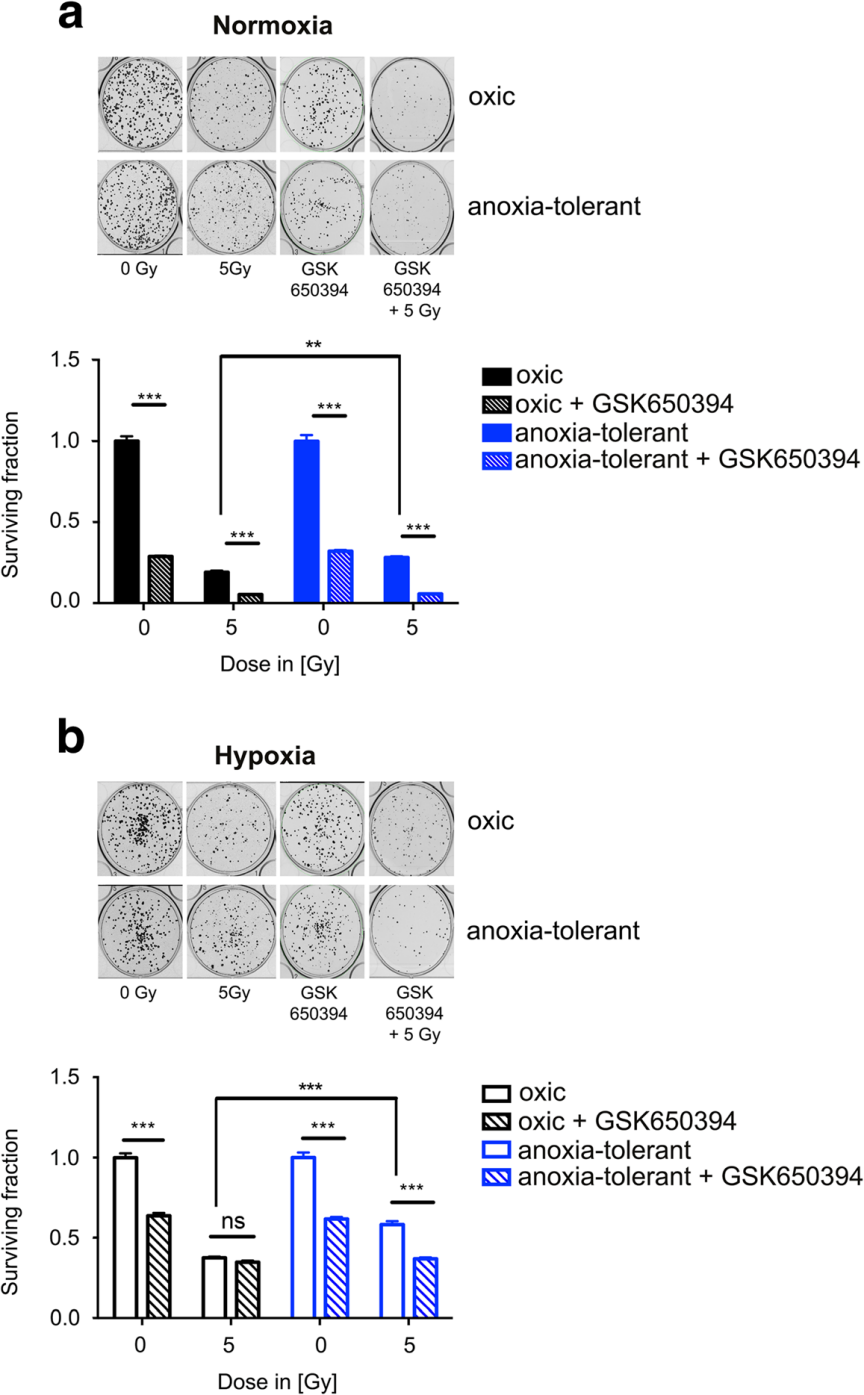
SGK1 inhibition decreases long-term survival and sensitizes to ionizing radiation. Colony formation of oxic and anoxia-tolerant NCI-H460 cells upon treatment with SGK1 inhibitor GSK650394 (5 μM), ionizing radiation (2 or 5 Gy), or the combination (pretreatment with GSK650394 for 2 h) under normoxic (20 % O_2_), **a**) and severely hypoxic conditions (0.2 % O_2_), **b**). Treatment in severe hypoxia was performed 2 h after pre-incubation in hypoxia. Upper panels show representative pictures of colonies formed after 10 days in normoxia (**a**) or severe hypoxia (**b**) as indicated. Values were normalized to the plating efficiency of the respective untreated cells. Lower panels represent quantification of the surviving fraction normalized to the plating efficiency of non-irradiated cells. Anoxia-tolerant NCI-H460 cells are characterized by increased radiation resistance as demonstrated by increased surviving fractions of anoxia-tolerant NCI-H460 cells compared to oxic controls upon exposure to ionizing radiation in normoxia (**a**) or hypoxia (**b**). SGK1 inhibition alone significantly reduced the number of clonogenic tumor cells in both cell lines. The effect was more prominent under normoxic conditions (**a**). (Pre-)treatment with GSK650394 was highly effective in reducing the surviving fraction of anoxia-tolerant NCI-H460 cells and potently sensitized both cell lines to the cytotoxic action of ionizing radiation under normoxic conditions (**a**). Combined treatment with ionizing radiation and GSK650394 under severely hypoxic conditions only decreased the numbers of clonogenic anoxia-tolerant cells but was almost without effect on the oxic control cells. Mean values ± SD are shown, *n* = 3. (* *p* ≤ 0.05, ** *p* < 0.01, *** *p* ≤ 0.001; two- way ANOVA with Bonferroni post-test). IR, ionizing radiation

To investigate whether we can exploit the dependency on SGK1 and FA uptake in a more clinical relevant setting for increasing sensitivity to radiotherapy, we next tested the effect of GSK650395 alone and in combination with ionizing radiation on long-term survival of oxic and anoxia-tolerant NCI-H460 cells (Fig. 4a, b).

**Fig. 5:**
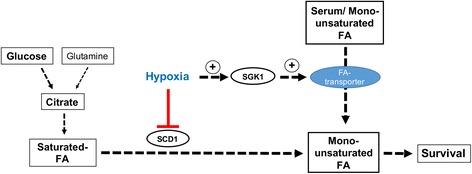
Model of SGK1´s role in FA uptake in hypoxia. In general, cells grown in normoxia generate (mono-)unsaturated FA from saturated stearic and palmitic acid by oxygen-dependent desaturation performed by SCD1. In normoxia, FA synthesis emerges predominantly from glucose metabolites and only to a little extent from glutamine. Blocking the process of oxygen-dependent desaturation by hypoxia-induced inhibition of SCD1 leads to increased dependence of cancer cells on the uptake of (mono-)unsaturated FA from the environment. We provide evidence for a role of SGK1 in FA uptake in NCI-H460 lung cancer cells exposed to severe hypoxia that may at least partially contribute to the radiosensitizing effect of SGK1-inhibition in anoxia-tolerant NCI-H460 cancer cells with increased resistance to radiation-induced apoptosis

As expected, the number of clonogenic cells declined in both cell lines with increasing radiation dose and significantly higher fractions of anoxia-tolerant cells survived compared to oxic controls particularly upon irradiation with 5 Gy under normoxic (Fig. 4a) and severely hypoxic conditions (Fig. 4b). Interestingly, treating the cells with GSK650394 largely decreased the number of clonogenic oxic and anoxia-tolerant cells under normoxic conditions (Fig. 4a). Combining ionizing radiation and GSK650394 resulted in almost complete eradication of clonogenic tumor cells in both cell lines (Fig. 4a). Treating the cells with GSK650394 alone decreased the number of clonogenic cells in both cell lines in severe hypoxia, but to a lesser extent compared to normoxia (Fig. 4b). Interestingly, under severely hypoxic conditions combined treatment with GSK650394 increased only the cytotoxic effect of ionizing radiation in anoxia-tolerant cancer cells but was almost without additional effect in the oxic NCI-H460 control cells (Fig. 4b). Our data reveal that SGK1-inhibiton is a promising approach for the eradication of normoxic, hypoxic and anoxia-tolerant cancer cells and is even able to sensitize anoxia-tolerant NCI-H460 cancer cells with increased resistance to radiation-induced cell death to the cytotoxic effects of ionizing radiation in normoxia and severe hypoxia.

## Discussion

There is a high need for new therapeutic approaches suited to overcome hypoxia-mediated therapy resistance. Here we show for the first time that SGK1 is important to FA uptake of NCI-H460 lung adenocarcinoma cells that becomes essential for cell survival under conditions of severe hypoxia. Interestingly, dependency on SGK1 and FA supply for cell survival in hypoxia was maintained in anoxia-tolerant NCI-H460 cells with increased resistance to radiation-induced cell death due to adaption to chronic cycling hypoxia-reoxygenation stress. Even more important, pharmacologic inhibition of SGK1 resulted in efficient eradication of clonogenic NCI-H460 cells when given alone or in combination with ionizing radiation, including anoxia-tolerant cells treated in severe hypoxia.

In more detail, we found that the toxic effect of serum deprivation on parental and anoxia-tolerant NCI-H460 cells could be rescued by supplementation with the mono-unsaturated FA oleic acid. These findings corroborate earlier observations on the dependency of A549 cancer cells on the uptake of unsaturated FA under hypoxic conditions [23]. The authors demonstrated that RAS-stimulated scavenging of serum FA instead of *de novo* lipogenesis from glucose rendered those cancer cells resistant to hypoxia as well to inhibitors of the oxygen-dependent enzyme SDC1 that operates in the glucose-dependent *de novo* lipogenesis pathway [21, 23, 42]. However, here we show in addition that dependency of RAS-driven NCI-H460 cells on exogenously added unsaturated FA and FA uptake for survival in hypoxia is maintained in an anoxia-tolerant cell variant that has been generated by chronic exposure to hypoxia-re-oxygenation stress [16]. Furthermore, we uncovered SGK1 as a regulator of FA uptake from a hypoxic tumor environment, thereby offering new options for the treatment of these highly radiation resistant cells (Fig. 5).

First hints to a potential role of kinases of the SGK1 family in FA uptake were provided by a study in yeast (*Saccharomyces cerevisiae*) [27]. The authors showed that the protein kinase YPK1, a yeast ortholog of the human kinase SGK1, was required for efficient FA uptake from the environment. Since the yeast ortholog of the human FA desaturase SCD1, OLE1, was only functionally active under conditions of sufficient oxygen supply, yeast were not able to generate unsaturated lipids from *de novo* synthesis in hypoxia. Thus, similar to the situation in human cells mentioned above, hypoxic yeast became dependent on increased FA uptake from their environment. Importantly, the deficiency in FA uptake in YPK1-deficient hypoxic yeast cells could be rescued by overexpression of human SGK1.

Here, we now provide evidence that human SGK1 plays a similar role in FA uptake and survival of hypoxic human cancer cells suggesting a functional conservation of YPK1/SGK1 in FA uptake in hypoxia. Our assumption is based on the following findings: i) we observed a pronounced up-regulation in the expression of SGK1 in response to acute severe hypoxia in parental and anoxia-tolerant NCI-H460 cancer cells; ii) Increased SGK1 expression in severe hypoxia correlated with a significant increase in the uptake of fluorescent FA in both cell lines; iii) Treatment with SGK1 inhibitor led to a significant decrease in FA uptake and this was associated with a pronounced eradication of hypoxic cancer cells.

The toxic effect of serum deprivation in severe hypoxia could be rescued in both cell lines by the addition of the unsaturated FA oleic acid; in contrast, the addition of the saturated FA palmitic acid was highly toxic. This finding supports the assumption that SCD1 is inhibited under severe hypoxia and is reminiscent of observations made in normoxic cancer cells exposed to pharmacological inhibition of SCD1 where the cells could only be rescued by unsaturated FA with *cis-*configuration such as oleic acid, whereas unsaturated FA in *trans-*configuration such as elaidic acid provided only a limited protection [18]. The authors of this study attributed this effect to the predominant *cis*-configuration of unsaturated FA in cellular membranes [18]. Furthermore, excessive accumulation of long-chain FA, particularly saturated FA such as palmitic acid, was shown to trigger cell stress and cellular dysfunction in rat neonatal cardiomyocytes, adipocytes, mouse embryonic fibroblasts and chinese hamster ovary cells even in normoxia [43–45]. In this context, excessive accumulation of saturated FA was shown to trigger the unfolded protein response (UPR), disruption of ER structure and cell death [44, 46]. Due to protective effect of desaturation in normoxia, SCD1 prevents the accumulation of cell-damaging lipid intermediates and thus an induction of apoptosis. However, this process is abrogated under hypoxia providing a potential explanation for the high toxicity of palmitic acid supplementation to serum-deprived NCI-H460 cells in severe hypoxia. On the other hand, triglycerides are able to bind palmitic acid to some extent, thereby preventing their pro-apoptotic effects [45, 47]. However, triglycerides generated in the presence of high amounts of palmitic acid are of minor value in cellular processes and may thus indirectly contribute to the so-called “lipid toxicity” of saturated FA [45]. As an example, triglycerides produced from unsaturated FA provide important building blocks for ceramide synthesis. The ability to incorporate oleic acid instead of palmitic acid into triglycerides may therefore explain why the addition of oleic acid together with palmitic acid abrogated the toxic effects of palmitic acid in severe hypoxia in our study. Altogether our observations underline the importance of supplementation and uptake of unsaturated FA for cell survival in hypoxia.

Interestingly, inhibition of SGK1 in serum-deprived NCI-H460 cells abrogated the observed rescue effect of oleic acid supplementation in severe hypoxia pointing to additional roles of SGK1 for cell survival apart from the regulation of FA uptake. This assumption is further supported by the observation that SGK1 inhibition also exerted toxic effects in normoxia. This is consistent with the recent findings that siRNA-induced down-regulation of SGK1 significantly reduced cell proliferation and survival in myeloma cells in normoxia [48].

We therefore speculate that SGK1 has additional roles for the survival of cancer cells. This highly conserved kinase is expressed in various tissue types in mammals and plays an important role in the cellular stress response. Of note SGK1 is involved in the regulation of membrane localization and function of various ion channels and nutrient transporters [31, 49–51]. For example, phosphorylation by SGK1 triggers activation and translocation of the glucose transporter GLUT1 to the plasma membrane [29, 52, 53]. In addition, SGK1 regulates various amino acid and peptide transporters responsible for the transport of nutrients (amino acids and oligopeptides) across the cellular membrane that are essential for cell proliferation and survival [31, 52–54]. Though SGK1 may not be required for maintenance of basic functions of ion channels and nutrient transporters [29, 31, 55], their SGK1-dependent regulation could become relevant under pathological conditions such as tumor growth. Here we add a novel aspect to the function of SGK1, namely regulation of FA uptake that was essential to the survival of cancer cells in severe hypoxia. We speculate that the role played by SGK1 in the regulation of nutrient uptake make the kinase an attractive therapeutic target for cancer cells with high metabolic demand, particularly in solid tumors with pronounced hypoxia.

In an effort to translate these novel findings in a more clinically relevant setting we finally demonstrated that inhibition of SGK1 is suited to increase the efficacy of ionizing radiation in NCI-H460 cells. Our results corroborate earlier in vitro findings on a significant increase in apoptosis and cell death of Caco-2 colon carcinoma cells by combined treatment with ionizing radiation and SGK1 inhibition [56]. Furthermore, SGK1 inhibition blocked tumor progression and had a radiosensitizing effect in a preclinical hepatocellular carcinoma (HCC) model in vivo and in vitro [57]. However, we demonstrate for the first time that SGK1-inhibition has also a potent radiosensitizing effect in a lung adenocarcinoma cell model in severe hypoxia, including anoxia-tolerant NCI-H460 cancer cells, and that this effect may at least partially be linked to reduced uptake of FA and other nutrients.

Interestingly, radiation itself was reported to trigger the up-regulated expression of SGK1 which together with other up-regulated proteins involved in proliferation and metabolic pathways promoted carcinogenesis in mouse mammary gland [58]. Furthermore, a recent study demonstrated that radiation changes the metabolite pattern in the tumor extracellular fluid as well as in the serum of irradiated cancer patients [59]. We speculate that SGK1 participates in stress-induced alterations in the transport of nutrients and metabolites across cellular membranes thereby promoting cell survival in cells exposed to severe hypoxia, ionizing radiation, or both. However, more detailed investigations are needed to unravel the exact mechanisms by which SGK1 increases cell survival and radioresistance to further explore SGK1 as therapeutic target in radiotherapy.

## Conclusions

Taken together our results demonstrate a key role of SGK1 in FA uptake and survival of NCI-H460 cancer cells in severe hypoxia and its use as therapeutic target for radio-sensitization. The finding that SGK1 inhibition was particularly effective in sensitizing radioresistant anoxia-tolerant NCI-H460 cancer cells to the cytotoxic effects of ionizing radiation under conditions of severe hypoxia provides new options for maximizing the effect of radiotherapy by pharmacological inhibition of molecular targets, like SGK1 that become essential to cancer cell survival in chronically hypoxic tumor fractions.

## Abbreviations

FA: fatty acid
GLUT1: glucose transporter 1
Hx: hypoxia
IR: ionizing radiation
Nx: normoxia
OA: oleic acid
OLE1: stearoyl-CoA desaturase (*Saccharomyces cerevisiae*)
PA: palmitic acid
PI: Propidium-iodide
qRT-PCR: quantitative real-time PCR
SCD1: stearoyl-CoA desaturase
SGK1: serum and glucocorticoid-inducible kinase 1
YPK1: Serine/threonine-protein kinase (*Saccharomyces cerevisiae*)
